# (Ethanolato)[2,3,7,8,12,13,17,18-octa­ethylporphyrinato(2−)]iron(III)

**DOI:** 10.1107/S1600536810041024

**Published:** 2010-10-23

**Authors:** Lin Cheng, Nan Xu, Douglas R. Powell, George B. Richter-Addo

**Affiliations:** a Department of Chemistry and Biochemistry, University of Oklahoma, 101 Stephenson Parkway, Norman, OK 73019-5251 USA

## Abstract

The title compound, [Fe(C_2_H_5_O)(C_36_H_44_N_4_)], contains a five-coordinate iron–porphyrin complex with an axial eth­oxy ligand. The iron(III) atom is displaced by 0.504 (2) Å towards the eth­oxy ligand from the 24-atom mean plane of the porphyrin. The average Fe—N_p_ distance is 2.078 (2) Å and the Fe—O distance is 1.8232 (18) Å. The eth­oxy C atoms are disordered in a 0.581 (12):0.419 (12) ratio. The bond angles of the Fe—O—C linkage are 128.6 (3) and 130.4 (3)°, respectively, for the major and minor occupancy C atoms.

## Related literature

For the structures of other related five-coordinate octa­ethyl­porphyrin iron(III) alkoxide complexes, see: Kanamori *et al.* (2005 ▶); Hatano & Uno (1990 ▶). Iron porphyrin alkoxide complexes can serve as structural models of tyrosinate ligated heme enzyme catalases which catalyze the degradation of hydrogen peroxide to water and oxygen, see: Chelikani *et al.* (2004 ▶).
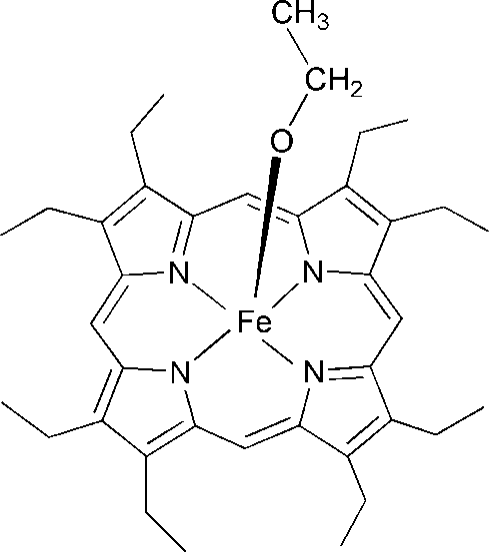


## Experimental

### 

#### Crystal data

[Fe(C_2_H_5_O)(C_36_H_44_N_4_)]
*M* *_r_* = 633.66
Triclinic, 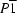

*a* = 10.3918 (13) Å
*b* = 10.4791 (12) Å
*c* = 16.4765 (18) Å
α = 106.312 (7)°
β = 105.181 (8)°
γ = 97.830 (9)°
*V* = 1618.7 (3) Å^3^
*Z* = 2
Mo *K*α radiation
μ = 0.50 mm^−1^
*T* = 163 K
0.48 × 0.36 × 0.28 mm

#### Data collection

Siemens P4 diffractometer
Absorption correction: ψ scan (North *et al.*, 1968 ▶) *T*_min_ = 0.794, *T*_max_ = 0.872
5868 measured reflections
5540 independent reflections
4779 reflections with *I* > 2σ(*I*)
*R*_int_ = 0.041
3 standard reflections every 97 reflections  intensity decay: 1.0%

#### Refinement

*R*[*F* ^2^ > 2σ(*F* ^2^)] = 0.045
*wR*(*F* ^2^) = 0.119
*S* = 1.06
5540 reflections
416 parameters
80 restraints
H-atom parameters constrained
Δρ_max_ = 0.80 e Å^−3^
Δρ_min_ = −0.69 e Å^−3^

### 

Data collection: *XSCANS* (Siemens, 1994 ▶); cell refinement: *XSCANS*; data reduction: *XSCANS*; program(s) used to solve structure: *SHELXTL* (Sheldrick, 2008 ▶); program(s) used to refine structure: *SHELXTL*; molecular graphics: *SHELXTL*; software used to prepare material for publication: *SHELXTL*.

## Supplementary Material

Crystal structure: contains datablocks I, global. DOI: 10.1107/S1600536810041024/fk2027sup1.cif

Structure factors: contains datablocks I. DOI: 10.1107/S1600536810041024/fk2027Isup2.hkl

Additional supplementary materials:  crystallographic information; 3D view; checkCIF report

## Figures and Tables

**Fig. 1.**
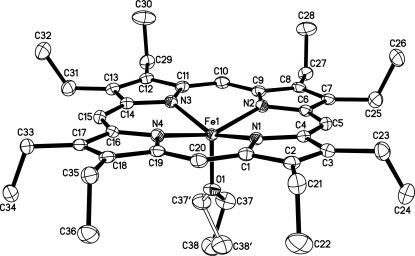
The molecular structure of (OEP)Fe(OEt). Displacement ellipsoids are drawn at the 50% probability level. H atoms are omitted for clarity.

## supplementary crystallographic information

### Comment

We report the structure of the five-coordinate (2,3,7,8,12,13,17,18-octaethylporphyrinato)(ethoxy)iron(III) complex (*i.e.*, (OEP)Fe(OEt)). Iron porphyrin alkoxide complexes can serve as structural models of tyrosinate ligated heme enzyme catalases which catalyze the degradation of hydrogen peroxide to water and oxygen (Chelikani *et al.* 2004). Other related octaethylporphyrin ferric alkoxide complexes have been synthesized and structurally characterized previously (Kanamori *et al.* 2005 and Hatano *et al.* 1990).

The ethoxy ligand was disordered at both carbon positions C37 and C38, and this O—C37—C38 fragment was modeled in two orientations. Occupancies for the disordered group refined to 0.581 (12) and 0.419 (12) for the unprimed and primed atoms, respectively. The molecular structure of (OEP)Fe(OEt) showing the disordered ethoxy group is shown in Figure 1. Selected bond lengths and angles are given in Table 1. The ethoxy group binds to the iron center through the O atom. The iron atom is displaced by 0.504 (2) Å from the 24-atom mean porphyrin plane toward the ethoxy ligand. The Fe—O distance of 1.8232 (13) Å is in the 1.816 (4) Å - 1.926 (3) Å range reported for similar octaethylporphyrin iron alkoxide complexes (Kanamori *et al.* 2005 and Hatano *et al.* 1990). The bond angles of the Fe—O—C linkage are 128.6 (3)° (for C37) and 130.4 (3)° (for C37'), respectively.

### Experimental

A suitable purple block-shaped crystal was grown unexpectedly by slow evaporation of a dichloromethane-ethanol (1:1) solution of µ-oxo-bis((octaethylporphyrinato)iron(III)) at room temperature under N_2_ under normal laboratory lighting.

### Refinement

H atoms were placed using assumed geometry with C—H(aromatic = 0.95 Å, methylene = 0.99 Å, and methyl = 0.98 Å). Displacement parameters of the H atoms were set to 1.2 (1.5 for methyl) times the isotropic equivalent for the bonded C.

### Crystal data

**Table:**

[Fe(C_2_H_5_O)(C_36_H_44_N_4_)]	*Z* = 2
*M**_r_* = 633.66	*F*(000) = 678
Triclinic, *P*1	*D*_x_ = 1.300 Mg m^−^^3^
Hall symbol: -P 1	Mo *K*α radiation, λ = 0.71073 Å
*a* = 10.3918 (13) Å	Cell parameters from 61 reflections
*b* = 10.4791 (12) Å	θ = 2.7–12.0°
*c* = 16.4765 (18) Å	µ = 0.50 mm^−^^1^
α = 106.312 (7)°	*T* = 163 K
β = 105.181 (8)°	Block, purple
γ = 97.830 (9)°	0.48 × 0.36 × 0.28 mm
*V* = 1618.7 (3) Å^3^	

### Data collection

**Table:**

Siemens P4 diffractometer	4779 reflections with *I* > 2σ(*I*)
Radiation source: fine-focus sealed tube	*R*_int_ = 0.041
graphite	θ_max_ = 25.0°, θ_min_ = 2.1°
ω scans	*h* = −12→12
Absorption correction: ψ scan (North *et al.*, 1968)	*k* = −11→11
*T*_min_ = 0.794, *T*_max_ = 0.872	*l* = −19→18
5868 measured reflections	3 standard reflections every 97 reflections
5540 independent reflections	intensity decay: 1.0%

### Refinement

**Table:**

Refinement on *F*^2^	Primary atom site location: structure-invariant direct methods
Least-squares matrix: full	Secondary atom site location: difference Fourier map
*R*[*F*^2^ > 2σ(*F*^2^)] = 0.045	Hydrogen site location: inferred from neighbouring sites
*wR*(*F*^2^) = 0.119	H-atom parameters constrained
*S* = 1.06	*w* = 1/[σ^2^(*F*_o_^2^) + (0.058*P*)^2^ + 1.240*P*] where *P* = (*F*_o_^2^ + 2*F*_c_^2^)/3
5540 reflections	(Δ/σ)_max_ < 0.001
416 parameters	Δρ_max_ = 0.80 e Å^−^^3^
80 restraints	Δρ_min_ = −0.69 e Å^−^^3^

### Special details

**Table:**

Refinement. Restraints: SADI 0.004 C37 C38 C37' C38' SADI 0.004 C37 O1 C37' O1 ISOR 0.008 o1 C37 C37' C38 C38' SIMU 0.008 o1 C37 C37' C38 C38'

### Fractional atomic coordinates and isotropic or equivalent isotropic displacement parameters (Å^2^)

**Table:**

	*x*	*y*	*z*	*U*_iso_*/*U*_eq_	Occ. (<1)
Fe1	−0.00859 (3)	0.13917 (3)	0.25182 (2)	0.01500 (12)	
O1	0.10667 (18)	0.11689 (18)	0.34875 (11)	0.0217 (4)	
N1	−0.1290 (2)	−0.0555 (2)	0.17747 (13)	0.0149 (4)	
N2	−0.1726 (2)	0.1803 (2)	0.29610 (14)	0.0165 (4)	
N3	0.0484 (2)	0.3473 (2)	0.27482 (14)	0.0178 (4)	
N4	0.0965 (2)	0.1113 (2)	0.15905 (13)	0.0172 (4)	
C1	−0.0962 (2)	−0.1555 (2)	0.11728 (16)	0.0169 (5)	
C2	−0.1904 (3)	−0.2852 (3)	0.09367 (16)	0.0184 (5)	
C3	−0.2814 (2)	−0.2613 (3)	0.13921 (16)	0.0182 (5)	
C4	−0.2412 (2)	−0.1181 (2)	0.19182 (16)	0.0160 (5)	
C5	−0.3064 (2)	−0.0531 (3)	0.24969 (17)	0.0183 (5)	
H5A	−0.3810	−0.1086	0.2560	0.022*	
C6	−0.2739 (2)	0.0847 (3)	0.29919 (16)	0.0176 (5)	
C7	−0.3451 (3)	0.1485 (3)	0.35864 (16)	0.0183 (5)	
C8	−0.2871 (2)	0.2847 (3)	0.38977 (16)	0.0183 (5)	
C9	−0.1802 (3)	0.3041 (3)	0.35071 (16)	0.0178 (5)	
C10	−0.0958 (3)	0.4286 (3)	0.36501 (17)	0.0193 (5)	
H10A	−0.1122	0.5074	0.4027	0.023*	
C11	0.0098 (3)	0.4494 (2)	0.32980 (16)	0.0181 (5)	
C12	0.0977 (3)	0.5803 (3)	0.34830 (17)	0.0192 (5)	
C13	0.1929 (3)	0.5556 (3)	0.30630 (17)	0.0210 (5)	
C14	0.1604 (3)	0.4108 (3)	0.25972 (17)	0.0185 (5)	
C15	0.2302 (3)	0.3439 (3)	0.20580 (17)	0.0202 (5)	
H15A	0.3072	0.3988	0.2015	0.024*	
C16	0.1999 (2)	0.2062 (3)	0.15776 (16)	0.0178 (5)	
C17	0.2702 (3)	0.1423 (3)	0.09768 (16)	0.0186 (5)	
C18	0.2061 (3)	0.0077 (3)	0.06180 (16)	0.0187 (5)	
C19	0.0986 (2)	−0.0115 (3)	0.10084 (16)	0.0171 (5)	
C20	0.0103 (3)	−0.1341 (3)	0.08288 (16)	0.0189 (5)	
H20A	0.0237	−0.2121	0.0427	0.023*	
C21	−0.1839 (3)	−0.4178 (3)	0.03267 (18)	0.0245 (6)	
H21A	−0.1475	−0.4008	−0.0141	0.029*	
H21B	−0.2776	−0.4760	0.0030	0.029*	
C22	−0.0940 (3)	−0.4932 (3)	0.0817 (2)	0.0394 (8)	
H22A	−0.0937	−0.5801	0.0394	0.059*	
H22B	−0.1300	−0.5108	0.1279	0.059*	
H22C	−0.0003	−0.4373	0.1095	0.059*	
C23	−0.4020 (3)	−0.3614 (3)	0.13609 (18)	0.0238 (6)	
H23A	−0.4249	−0.4420	0.0818	0.029*	
H23B	−0.4818	−0.3186	0.1314	0.029*	
C24	−0.3786 (3)	−0.4091 (3)	0.2169 (2)	0.0325 (7)	
H24A	−0.4612	−0.4738	0.2104	0.049*	
H24B	−0.3582	−0.3303	0.2709	0.049*	
H24C	−0.3014	−0.4539	0.2212	0.049*	
C25	−0.4630 (3)	0.0755 (3)	0.37693 (18)	0.0238 (6)	
H25A	−0.4627	0.1247	0.4378	0.029*	
H25B	−0.4510	−0.0174	0.3747	0.029*	
C26	−0.6005 (3)	0.0646 (3)	0.3107 (2)	0.0316 (7)	
H26A	−0.6737	0.0135	0.3242	0.047*	
H26B	−0.6011	0.0170	0.2501	0.047*	
H26C	−0.6153	0.1563	0.3150	0.047*	
C27	−0.3249 (3)	0.3972 (3)	0.45254 (17)	0.0221 (6)	
H27A	−0.2403	0.4652	0.4929	0.027*	
H27B	−0.3688	0.3583	0.4896	0.027*	
C28	−0.4215 (3)	0.4691 (3)	0.4049 (2)	0.0322 (7)	
H28A	−0.4383	0.5441	0.4491	0.048*	
H28B	−0.5084	0.4039	0.3683	0.048*	
H28C	−0.3800	0.5056	0.3667	0.048*	
C29	0.0843 (3)	0.7159 (3)	0.40301 (18)	0.0243 (6)	
H29A	0.1767	0.7762	0.4347	0.029*	
H29B	0.0424	0.7027	0.4484	0.029*	
C30	−0.0017 (4)	0.7849 (3)	0.3473 (2)	0.0410 (8)	
H30A	−0.0097	0.8714	0.3861	0.061*	
H30B	−0.0930	0.7253	0.3152	0.061*	
H30C	0.0419	0.8028	0.3043	0.061*	
C31	0.3119 (3)	0.6568 (3)	0.30812 (19)	0.0261 (6)	
H31A	0.3939	0.6176	0.3164	0.031*	
H31B	0.3309	0.7402	0.3601	0.031*	
C32	0.2888 (3)	0.6967 (3)	0.2244 (2)	0.0324 (7)	
H32A	0.3703	0.7619	0.2299	0.049*	
H32B	0.2098	0.7388	0.2168	0.049*	
H32C	0.2713	0.6151	0.1725	0.049*	
C33	0.3916 (3)	0.2127 (3)	0.08191 (17)	0.0218 (6)	
H33A	0.3837	0.3070	0.0854	0.026*	
H33B	0.3923	0.1644	0.0211	0.026*	
C34	0.5269 (3)	0.2175 (3)	0.14961 (19)	0.0280 (6)	
H34A	0.6031	0.2683	0.1385	0.042*	
H34B	0.5383	0.1243	0.1435	0.042*	
H34C	0.5258	0.2629	0.2101	0.042*	
C35	0.2387 (3)	−0.1034 (3)	−0.00465 (17)	0.0226 (6)	
H35A	0.2826	−0.0629	−0.0410	0.027*	
H35B	0.1525	−0.1687	−0.0452	0.027*	
C36	0.3337 (3)	−0.1801 (3)	0.04001 (19)	0.0288 (6)	
H36A	0.3506	−0.2526	−0.0057	0.043*	
H36B	0.2908	−0.2204	0.0761	0.043*	
H36C	0.4207	−0.1166	0.0783	0.043*	
C37	0.1180 (8)	0.1737 (7)	0.4386 (3)	0.0313 (15)	0.581 (12)
H37A	0.0361	0.1321	0.4501	0.038*	0.581 (12)
H37B	0.1241	0.2732	0.4539	0.038*	0.581 (12)
C38	0.2486 (12)	0.1468 (16)	0.4969 (9)	0.037 (3)	0.581 (12)
H38A	0.2590	0.1891	0.5600	0.055*	0.581 (12)
H38B	0.3290	0.1860	0.4840	0.055*	0.581 (12)
H38C	0.2402	0.0482	0.4833	0.055*	0.581 (12)
C37'	0.1811 (10)	0.2115 (7)	0.4323 (4)	0.0301 (19)	0.419 (12)
H37C	0.1254	0.2757	0.4525	0.036*	0.419 (12)
H37D	0.2649	0.2647	0.4292	0.036*	0.419 (12)
C38'	0.2198 (19)	0.132 (2)	0.4987 (13)	0.038 (4)	0.419 (12)
H38D	0.2624	0.1973	0.5593	0.057*	0.419 (12)
H38E	0.2843	0.0778	0.4825	0.057*	0.419 (12)
H38F	0.1371	0.0719	0.4962	0.057*	0.419 (12)

### Atomic displacement parameters (Å^2^)

**Table:**

	*U*^11^	*U*^22^	*U*^33^	*U*^12^	*U*^13^	*U*^23^
Fe1	0.0155 (2)	0.0140 (2)	0.0194 (2)	0.00698 (14)	0.00862 (14)	0.00698 (14)
O1	0.0239 (9)	0.0237 (10)	0.0201 (9)	0.0128 (8)	0.0064 (7)	0.0081 (7)
N1	0.0155 (10)	0.0147 (10)	0.0173 (10)	0.0066 (8)	0.0070 (8)	0.0062 (8)
N2	0.0160 (10)	0.0157 (11)	0.0221 (11)	0.0078 (8)	0.0097 (9)	0.0072 (9)
N3	0.0179 (11)	0.0168 (11)	0.0237 (11)	0.0086 (9)	0.0098 (9)	0.0093 (9)
N4	0.0175 (11)	0.0176 (11)	0.0207 (11)	0.0075 (9)	0.0094 (9)	0.0081 (9)
C1	0.0181 (12)	0.0156 (12)	0.0171 (12)	0.0062 (10)	0.0045 (10)	0.0052 (10)
C2	0.0183 (13)	0.0181 (13)	0.0176 (12)	0.0056 (10)	0.0020 (10)	0.0068 (10)
C3	0.0155 (12)	0.0194 (13)	0.0201 (13)	0.0049 (10)	0.0039 (10)	0.0084 (10)
C4	0.0154 (12)	0.0171 (13)	0.0184 (12)	0.0060 (10)	0.0051 (10)	0.0091 (10)
C5	0.0157 (12)	0.0202 (13)	0.0239 (13)	0.0056 (10)	0.0089 (10)	0.0116 (11)
C6	0.0158 (12)	0.0224 (13)	0.0207 (13)	0.0092 (10)	0.0082 (10)	0.0116 (10)
C7	0.0177 (13)	0.0237 (14)	0.0194 (12)	0.0109 (11)	0.0081 (10)	0.0109 (10)
C8	0.0171 (12)	0.0227 (14)	0.0192 (13)	0.0116 (11)	0.0067 (10)	0.0090 (10)
C9	0.0191 (13)	0.0191 (13)	0.0193 (12)	0.0107 (10)	0.0078 (10)	0.0081 (10)
C10	0.0230 (13)	0.0160 (13)	0.0219 (13)	0.0118 (11)	0.0086 (11)	0.0058 (10)
C11	0.0206 (13)	0.0139 (12)	0.0211 (13)	0.0069 (10)	0.0058 (10)	0.0071 (10)
C12	0.0219 (13)	0.0172 (13)	0.0209 (13)	0.0077 (10)	0.0057 (10)	0.0091 (10)
C13	0.0222 (14)	0.0199 (14)	0.0238 (13)	0.0068 (11)	0.0064 (11)	0.0117 (11)
C14	0.0175 (13)	0.0186 (13)	0.0228 (13)	0.0072 (10)	0.0061 (10)	0.0106 (10)
C15	0.0182 (13)	0.0218 (14)	0.0267 (14)	0.0062 (10)	0.0105 (11)	0.0135 (11)
C16	0.0145 (12)	0.0232 (14)	0.0222 (13)	0.0089 (10)	0.0078 (10)	0.0132 (11)
C17	0.0184 (13)	0.0245 (14)	0.0200 (13)	0.0113 (11)	0.0079 (10)	0.0131 (11)
C18	0.0214 (13)	0.0226 (14)	0.0181 (12)	0.0121 (11)	0.0074 (10)	0.0116 (10)
C19	0.0187 (12)	0.0202 (13)	0.0168 (12)	0.0113 (10)	0.0063 (10)	0.0090 (10)
C20	0.0212 (13)	0.0174 (13)	0.0200 (13)	0.0092 (10)	0.0074 (10)	0.0062 (10)
C21	0.0255 (14)	0.0189 (14)	0.0264 (14)	0.0034 (11)	0.0111 (12)	0.0014 (11)
C22	0.0398 (18)	0.0248 (16)	0.050 (2)	0.0166 (14)	0.0109 (15)	0.0055 (14)
C23	0.0203 (13)	0.0201 (14)	0.0277 (14)	0.0017 (11)	0.0069 (11)	0.0050 (11)
C24	0.0316 (16)	0.0277 (16)	0.0403 (17)	0.0008 (13)	0.0121 (13)	0.0162 (13)
C25	0.0252 (14)	0.0258 (14)	0.0262 (14)	0.0096 (11)	0.0152 (12)	0.0090 (11)
C26	0.0213 (14)	0.0356 (17)	0.0438 (18)	0.0088 (12)	0.0149 (13)	0.0168 (14)
C27	0.0269 (14)	0.0236 (14)	0.0221 (13)	0.0124 (11)	0.0133 (11)	0.0087 (11)
C28	0.0407 (17)	0.0339 (17)	0.0325 (16)	0.0269 (14)	0.0172 (14)	0.0130 (13)
C29	0.0286 (15)	0.0154 (13)	0.0267 (14)	0.0048 (11)	0.0082 (12)	0.0045 (11)
C30	0.049 (2)	0.0264 (17)	0.0412 (18)	0.0202 (15)	0.0016 (15)	0.0072 (14)
C31	0.0280 (15)	0.0197 (14)	0.0320 (15)	0.0043 (11)	0.0109 (12)	0.0099 (12)
C32	0.0340 (16)	0.0303 (16)	0.0368 (17)	0.0048 (13)	0.0139 (13)	0.0157 (13)
C33	0.0206 (13)	0.0281 (15)	0.0247 (14)	0.0097 (11)	0.0125 (11)	0.0140 (11)
C34	0.0204 (14)	0.0388 (17)	0.0303 (15)	0.0082 (12)	0.0102 (12)	0.0172 (13)
C35	0.0257 (14)	0.0277 (15)	0.0216 (13)	0.0126 (12)	0.0136 (11)	0.0103 (11)
C36	0.0326 (16)	0.0276 (15)	0.0323 (15)	0.0166 (13)	0.0147 (13)	0.0103 (12)
C37	0.035 (3)	0.032 (3)	0.024 (2)	0.013 (2)	0.009 (2)	0.0019 (19)
C38	0.036 (5)	0.045 (4)	0.023 (3)	0.008 (4)	0.003 (3)	0.008 (3)
C37'	0.030 (4)	0.030 (3)	0.025 (3)	0.007 (3)	0.003 (3)	0.007 (2)
C38'	0.038 (6)	0.040 (5)	0.025 (4)	0.005 (4)	0.001 (4)	0.007 (3)

### Geometric parameters (Å, °)

**Table:**

Fe1—O1	1.8232 (18)	C23—H23A	0.9900
Fe1—N2	2.074 (2)	C23—H23B	0.9900
Fe1—N3	2.078 (2)	C24—H24A	0.9800
Fe1—N1	2.080 (2)	C24—H24B	0.9800
Fe1—N4	2.080 (2)	C24—H24C	0.9800
O1—C37'	1.394 (5)	C25—C26	1.522 (4)
O1—C37	1.398 (4)	C25—H25A	0.9900
N1—C4	1.367 (3)	C25—H25B	0.9900
N1—C1	1.374 (3)	C26—H26A	0.9800
N2—C6	1.372 (3)	C26—H26B	0.9800
N2—C9	1.381 (3)	C26—H26C	0.9800
N3—C11	1.374 (3)	C27—C28	1.522 (4)
N3—C14	1.376 (3)	C27—H27A	0.9900
N4—C16	1.369 (3)	C27—H27B	0.9900
N4—C19	1.379 (3)	C28—H28A	0.9800
C1—C20	1.389 (3)	C28—H28B	0.9800
C1—C2	1.448 (4)	C28—H28C	0.9800
C2—C3	1.360 (4)	C29—C30	1.511 (4)
C2—C21	1.491 (3)	C29—H29A	0.9900
C3—C4	1.445 (3)	C29—H29B	0.9900
C3—C23	1.500 (3)	C30—H30A	0.9800
C4—C5	1.390 (3)	C30—H30B	0.9800
C5—C6	1.387 (4)	C30—H30C	0.9800
C5—H5A	0.9500	C31—C32	1.521 (4)
C6—C7	1.449 (3)	C31—H31A	0.9900
C7—C8	1.362 (4)	C31—H31B	0.9900
C7—C25	1.499 (4)	C32—H32A	0.9800
C8—C9	1.441 (3)	C32—H32B	0.9800
C8—C27	1.506 (3)	C32—H32C	0.9800
C9—C10	1.390 (4)	C33—C34	1.533 (4)
C10—C11	1.389 (4)	C33—H33A	0.9900
C10—H10A	0.9500	C33—H33B	0.9900
C11—C12	1.444 (4)	C34—H34A	0.9800
C12—C13	1.361 (4)	C34—H34B	0.9800
C12—C29	1.501 (3)	C34—H34C	0.9800
C13—C14	1.441 (4)	C35—C36	1.526 (4)
C13—C31	1.503 (4)	C35—H35A	0.9900
C14—C15	1.391 (4)	C35—H35B	0.9900
C15—C16	1.383 (4)	C36—H36A	0.9800
C15—H15A	0.9500	C36—H36B	0.9800
C16—C17	1.450 (3)	C36—H36C	0.9800
C17—C18	1.361 (4)	C37—C38	1.554 (7)
C17—C33	1.493 (3)	C37—H37A	0.9900
C18—C19	1.446 (3)	C37—H37B	0.9900
C18—C35	1.502 (3)	C38—H38A	0.9800
C19—C20	1.379 (4)	C38—H38B	0.9800
C20—H20A	0.9500	C38—H38C	0.9800
C21—C22	1.522 (4)	C37'—C38'	1.555 (7)
C21—H21A	0.9900	C37'—H37C	0.9900
C21—H21B	0.9900	C37'—H37D	0.9900
C22—H22A	0.9800	C38'—H38D	0.9800
C22—H22B	0.9800	C38'—H38E	0.9800
C22—H22C	0.9800	C38'—H38F	0.9800
C23—C24	1.523 (4)		
			
O1—Fe1—N2	101.65 (8)	C23—C24—H24A	109.5
O1—Fe1—N3	103.00 (8)	C23—C24—H24B	109.5
N2—Fe1—N3	86.77 (8)	H24A—C24—H24B	109.5
O1—Fe1—N1	104.41 (8)	C23—C24—H24C	109.5
N2—Fe1—N1	87.11 (8)	H24A—C24—H24C	109.5
N3—Fe1—N1	152.58 (8)	H24B—C24—H24C	109.5
O1—Fe1—N4	104.19 (8)	C7—C25—C26	112.7 (2)
N2—Fe1—N4	154.16 (8)	C7—C25—H25A	109.1
N3—Fe1—N4	87.00 (8)	C26—C25—H25A	109.1
N1—Fe1—N4	86.97 (8)	C7—C25—H25B	109.1
C37'—O1—Fe1	130.4 (3)	C26—C25—H25B	109.1
C37—O1—Fe1	128.6 (3)	H25A—C25—H25B	107.8
C4—N1—C1	106.0 (2)	C25—C26—H26A	109.5
C4—N1—Fe1	126.34 (16)	C25—C26—H26B	109.5
C1—N1—Fe1	126.48 (16)	H26A—C26—H26B	109.5
C6—N2—C9	105.7 (2)	C25—C26—H26C	109.5
C6—N2—Fe1	125.44 (16)	H26A—C26—H26C	109.5
C9—N2—Fe1	126.34 (16)	H26B—C26—H26C	109.5
C11—N3—C14	105.7 (2)	C8—C27—C28	113.3 (2)
C11—N3—Fe1	126.62 (16)	C8—C27—H27A	108.9
C14—N3—Fe1	125.25 (16)	C28—C27—H27A	108.9
C16—N4—C19	106.0 (2)	C8—C27—H27B	108.9
C16—N4—Fe1	125.94 (17)	C28—C27—H27B	108.9
C19—N4—Fe1	126.59 (16)	H27A—C27—H27B	107.7
N1—C1—C20	124.6 (2)	C27—C28—H28A	109.5
N1—C1—C2	110.2 (2)	C27—C28—H28B	109.5
C20—C1—C2	125.2 (2)	H28A—C28—H28B	109.5
C3—C2—C1	106.6 (2)	C27—C28—H28C	109.5
C3—C2—C21	127.9 (2)	H28A—C28—H28C	109.5
C1—C2—C21	125.5 (2)	H28B—C28—H28C	109.5
C2—C3—C4	106.7 (2)	C12—C29—C30	112.6 (2)
C2—C3—C23	127.8 (2)	C12—C29—H29A	109.1
C4—C3—C23	125.5 (2)	C30—C29—H29A	109.1
N1—C4—C5	124.6 (2)	C12—C29—H29B	109.1
N1—C4—C3	110.5 (2)	C30—C29—H29B	109.1
C5—C4—C3	124.8 (2)	H29A—C29—H29B	107.8
C6—C5—C4	126.7 (2)	C29—C30—H30A	109.5
C6—C5—H5A	116.7	C29—C30—H30B	109.5
C4—C5—H5A	116.7	H30A—C30—H30B	109.5
N2—C6—C5	124.5 (2)	C29—C30—H30C	109.5
N2—C6—C7	110.6 (2)	H30A—C30—H30C	109.5
C5—C6—C7	124.9 (2)	H30B—C30—H30C	109.5
C8—C7—C6	106.4 (2)	C13—C31—C32	113.9 (2)
C8—C7—C25	128.4 (2)	C13—C31—H31A	108.8
C6—C7—C25	125.2 (2)	C32—C31—H31A	108.8
C7—C8—C9	107.1 (2)	C13—C31—H31B	108.8
C7—C8—C27	127.9 (2)	C32—C31—H31B	108.8
C9—C8—C27	124.9 (2)	H31A—C31—H31B	107.7
N2—C9—C10	124.1 (2)	C31—C32—H32A	109.5
N2—C9—C8	110.2 (2)	C31—C32—H32B	109.5
C10—C9—C8	125.6 (2)	H32A—C32—H32B	109.5
C11—C10—C9	126.7 (2)	C31—C32—H32C	109.5
C11—C10—H10A	116.7	H32A—C32—H32C	109.5
C9—C10—H10A	116.7	H32B—C32—H32C	109.5
N3—C11—C10	124.5 (2)	C17—C33—C34	112.4 (2)
N3—C11—C12	110.5 (2)	C17—C33—H33A	109.1
C10—C11—C12	125.0 (2)	C34—C33—H33A	109.1
C13—C12—C11	106.6 (2)	C17—C33—H33B	109.1
C13—C12—C29	127.4 (2)	C34—C33—H33B	109.1
C11—C12—C29	126.0 (2)	H33A—C33—H33B	107.9
C12—C13—C14	106.8 (2)	C33—C34—H34A	109.5
C12—C13—C31	127.8 (2)	C33—C34—H34B	109.5
C14—C13—C31	125.4 (2)	H34A—C34—H34B	109.5
N3—C14—C15	124.4 (2)	C33—C34—H34C	109.5
N3—C14—C13	110.4 (2)	H34A—C34—H34C	109.5
C15—C14—C13	125.2 (2)	H34B—C34—H34C	109.5
C16—C15—C14	127.1 (2)	C18—C35—C36	112.3 (2)
C16—C15—H15A	116.5	C18—C35—H35A	109.1
C14—C15—H15A	116.5	C36—C35—H35A	109.1
N4—C16—C15	124.4 (2)	C18—C35—H35B	109.1
N4—C16—C17	110.5 (2)	C36—C35—H35B	109.1
C15—C16—C17	125.1 (2)	H35A—C35—H35B	107.9
C18—C17—C16	106.5 (2)	C35—C36—H36A	109.5
C18—C17—C33	127.9 (2)	C35—C36—H36B	109.5
C16—C17—C33	125.6 (2)	H36A—C36—H36B	109.5
C17—C18—C19	107.0 (2)	C35—C36—H36C	109.5
C17—C18—C35	128.0 (2)	H36A—C36—H36C	109.5
C19—C18—C35	125.0 (2)	H36B—C36—H36C	109.5
N4—C19—C20	124.5 (2)	O1—C37—C38	109.0 (7)
N4—C19—C18	110.0 (2)	O1—C37—H37A	109.9
C20—C19—C18	125.4 (2)	C38—C37—H37A	109.9
C19—C20—C1	126.9 (2)	O1—C37—H37B	109.9
C19—C20—H20A	116.5	C38—C37—H37B	109.9
C1—C20—H20A	116.5	H37A—C37—H37B	108.3
C2—C21—C22	112.2 (2)	C37—C38—H38A	109.5
C2—C21—H21A	109.2	C37—C38—H38B	109.5
C22—C21—H21A	109.2	H38A—C38—H38B	109.5
C2—C21—H21B	109.2	C37—C38—H38C	109.5
C22—C21—H21B	109.2	H38A—C38—H38C	109.5
H21A—C21—H21B	107.9	H38B—C38—H38C	109.5
C21—C22—H22A	109.5	O1—C37'—C38'	108.2 (10)
C21—C22—H22B	109.5	O1—C37'—H37C	110.1
H22A—C22—H22B	109.5	C38'—C37'—H37C	110.1
C21—C22—H22C	109.5	O1—C37'—H37D	110.1
H22A—C22—H22C	109.5	C38'—C37'—H37D	110.1
H22B—C22—H22C	109.5	H37C—C37'—H37D	108.4
C3—C23—C24	113.9 (2)	C37'—C38'—H38D	109.5
C3—C23—H23A	108.8	C37'—C38'—H38E	109.5
C24—C23—H23A	108.8	H38D—C38'—H38E	109.5
C3—C23—H23B	108.8	C37'—C38'—H38F	109.5
C24—C23—H23B	108.8	H38D—C38'—H38F	109.5
H23A—C23—H23B	107.7	H38E—C38'—H38F	109.5
			
N2—Fe1—O1—C37'	−69.0 (7)	Fe1—N2—C9—C10	18.3 (3)
N3—Fe1—O1—C37'	20.4 (7)	C6—N2—C9—C8	0.9 (3)
N1—Fe1—O1—C37'	−159.0 (7)	Fe1—N2—C9—C8	−161.78 (16)
N4—Fe1—O1—C37'	110.5 (7)	C7—C8—C9—N2	−0.1 (3)
N2—Fe1—O1—C37	−27.4 (5)	C27—C8—C9—N2	−179.7 (2)
N3—Fe1—O1—C37	61.9 (5)	C7—C8—C9—C10	179.8 (2)
N1—Fe1—O1—C37	−117.4 (5)	C27—C8—C9—C10	0.2 (4)
N4—Fe1—O1—C37	152.1 (5)	N2—C9—C10—C11	−1.7 (4)
O1—Fe1—N1—C4	81.3 (2)	C8—C9—C10—C11	178.4 (2)
N2—Fe1—N1—C4	−20.02 (19)	C14—N3—C11—C10	−176.7 (2)
N3—Fe1—N1—C4	−97.3 (2)	Fe1—N3—C11—C10	−13.9 (4)
N4—Fe1—N1—C4	−174.9 (2)	C14—N3—C11—C12	1.2 (3)
O1—Fe1—N1—C1	−84.3 (2)	Fe1—N3—C11—C12	163.94 (16)
N2—Fe1—N1—C1	174.4 (2)	C9—C10—C11—N3	−0.6 (4)
N3—Fe1—N1—C1	97.1 (2)	C9—C10—C11—C12	−178.2 (2)
N4—Fe1—N1—C1	19.6 (2)	N3—C11—C12—C13	−2.0 (3)
O1—Fe1—N2—C6	−79.7 (2)	C10—C11—C12—C13	175.8 (2)
N3—Fe1—N2—C6	177.6 (2)	N3—C11—C12—C29	177.7 (2)
N1—Fe1—N2—C6	24.4 (2)	C10—C11—C12—C29	−4.5 (4)
N4—Fe1—N2—C6	101.3 (2)	C11—C12—C13—C14	2.0 (3)
O1—Fe1—N2—C9	79.7 (2)	C29—C12—C13—C14	−177.7 (2)
N3—Fe1—N2—C9	−22.9 (2)	C11—C12—C13—C31	−176.9 (2)
N1—Fe1—N2—C9	−176.2 (2)	C29—C12—C13—C31	3.4 (4)
N4—Fe1—N2—C9	−99.2 (3)	C11—N3—C14—C15	−178.9 (2)
O1—Fe1—N3—C11	−80.3 (2)	Fe1—N3—C14—C15	18.0 (3)
N2—Fe1—N3—C11	20.9 (2)	C11—N3—C14—C13	0.1 (3)
N1—Fe1—N3—C11	98.3 (2)	Fe1—N3—C14—C13	−163.00 (16)
N4—Fe1—N3—C11	175.8 (2)	C12—C13—C14—N3	−1.3 (3)
O1—Fe1—N3—C14	79.2 (2)	C31—C13—C14—N3	177.6 (2)
N2—Fe1—N3—C14	−179.6 (2)	C12—C13—C14—C15	177.6 (2)
N1—Fe1—N3—C14	−102.2 (2)	C31—C13—C14—C15	−3.4 (4)
N4—Fe1—N3—C14	−24.6 (2)	N3—C14—C15—C16	1.1 (4)
O1—Fe1—N4—C16	−79.3 (2)	C13—C14—C15—C16	−177.8 (2)
N2—Fe1—N4—C16	99.6 (2)	C19—N4—C16—C15	178.4 (2)
N3—Fe1—N4—C16	23.3 (2)	Fe1—N4—C16—C15	−14.9 (3)
N1—Fe1—N4—C16	176.6 (2)	C19—N4—C16—C17	−0.3 (3)
O1—Fe1—N4—C19	84.8 (2)	Fe1—N4—C16—C17	166.46 (16)
N2—Fe1—N4—C19	−96.3 (2)	C14—C15—C16—N4	−2.8 (4)
N3—Fe1—N4—C19	−172.6 (2)	C14—C15—C16—C17	175.7 (2)
N1—Fe1—N4—C19	−19.3 (2)	N4—C16—C17—C18	0.9 (3)
C4—N1—C1—C20	178.3 (2)	C15—C16—C17—C18	−177.8 (2)
Fe1—N1—C1—C20	−13.7 (3)	N4—C16—C17—C33	−177.4 (2)
C4—N1—C1—C2	−0.4 (3)	C15—C16—C17—C33	3.9 (4)
Fe1—N1—C1—C2	167.51 (16)	C16—C17—C18—C19	−1.0 (3)
N1—C1—C2—C3	1.0 (3)	C33—C17—C18—C19	177.3 (2)
C20—C1—C2—C3	−177.7 (2)	C16—C17—C18—C35	179.9 (2)
N1—C1—C2—C21	−177.7 (2)	C33—C17—C18—C35	−1.9 (4)
C20—C1—C2—C21	3.6 (4)	C16—N4—C19—C20	179.8 (2)
C1—C2—C3—C4	−1.1 (3)	Fe1—N4—C19—C20	13.1 (3)
C21—C2—C3—C4	177.5 (2)	C16—N4—C19—C18	−0.3 (3)
C1—C2—C3—C23	178.2 (2)	Fe1—N4—C19—C18	−166.99 (16)
C21—C2—C3—C23	−3.2 (4)	C17—C18—C19—N4	0.9 (3)
C1—N1—C4—C5	179.1 (2)	C35—C18—C19—N4	−180.0 (2)
Fe1—N1—C4—C5	11.2 (3)	C17—C18—C19—C20	−179.3 (2)
C1—N1—C4—C3	−0.3 (3)	C35—C18—C19—C20	−0.1 (4)
Fe1—N1—C4—C3	−168.24 (16)	N4—C19—C20—C1	1.7 (4)
C2—C3—C4—N1	0.9 (3)	C18—C19—C20—C1	−178.2 (2)
C23—C3—C4—N1	−178.4 (2)	N1—C1—C20—C19	−1.4 (4)
C2—C3—C4—C5	−178.5 (2)	C2—C1—C20—C19	177.2 (2)
C23—C3—C4—C5	2.2 (4)	C3—C2—C21—C22	−90.1 (3)
N1—C4—C5—C6	2.8 (4)	C1—C2—C21—C22	88.4 (3)
C3—C4—C5—C6	−177.8 (2)	C2—C3—C23—C24	103.6 (3)
C9—N2—C6—C5	176.4 (2)	C4—C3—C23—C24	−77.3 (3)
Fe1—N2—C6—C5	−20.7 (3)	C8—C7—C25—C26	89.3 (3)
C9—N2—C6—C7	−1.4 (3)	C6—C7—C25—C26	−87.7 (3)
Fe1—N2—C6—C7	161.52 (16)	C7—C8—C27—C28	−96.2 (3)
C4—C5—C6—N2	2.3 (4)	C9—C8—C27—C28	83.3 (3)
C4—C5—C6—C7	179.8 (2)	C13—C12—C29—C30	87.5 (3)
N2—C6—C7—C8	1.3 (3)	C11—C12—C29—C30	−92.2 (3)
C5—C6—C7—C8	−176.5 (2)	C12—C13—C31—C32	−102.6 (3)
N2—C6—C7—C25	178.9 (2)	C14—C13—C31—C32	78.7 (3)
C5—C6—C7—C25	1.1 (4)	C18—C17—C33—C34	−91.6 (3)
C6—C7—C8—C9	−0.7 (3)	C16—C17—C33—C34	86.4 (3)
C25—C7—C8—C9	−178.1 (2)	C17—C18—C35—C36	94.2 (3)
C6—C7—C8—C27	178.9 (2)	C19—C18—C35—C36	−84.7 (3)
C25—C7—C8—C27	1.5 (4)	Fe1—O1—C37—C38	−169.3 (6)
C6—N2—C9—C10	−179.0 (2)	Fe1—O1—C37'—C38'	160.2 (9)
